# Poly[diaqua­(μ_3_-pyridine-3,5-dicarboxyl­ato-κ^3^ *N*:*O* ^3^:*O* ^5^)copper(II)]

**DOI:** 10.1107/S1600536809013889

**Published:** 2009-04-22

**Authors:** Lin Du, Li-Nan Li, Qi-Hua Zhao

**Affiliations:** a School of Chemical Science and Technology, Key Laboratory of Medicinal Chemistry for Natural Resources, Ministry of Education, Yunnan University, Kunming 650091, People’s Republic of China

## Abstract

The title complex, [Cu(C_7_H_3_NO_4_)(H_2_O)_2_]_*n*_, was prepared under hydro­thermal reaction conditions. In the crystal structure, the Cu^II^ cation is located on a twofold rotation axis and is coordinated by two carboxyl­ate O atoms and one N atom from three pyridine-3,5-dicarboxyl­ate (PDA) anions and two water mol­ecules with a distorted trigonal–bipyramidal geometry. The tridentate PDA anion is also located on the twofold rotation axis and bridges the Cu^II^ cations to form a two-dimensional polymeric layer. O—H⋯O hydrogen bonding between layers links the two-dimensional layers into a three-dimensional supra­molecular framework.

## Related literature

For background, see: Chang *et al.* (2005 ▶); Hou *et al.* (2004 ▶). For related structures, see: Plater *et al.* (1998 ▶); Whitfield *et al.* (2001 ▶).
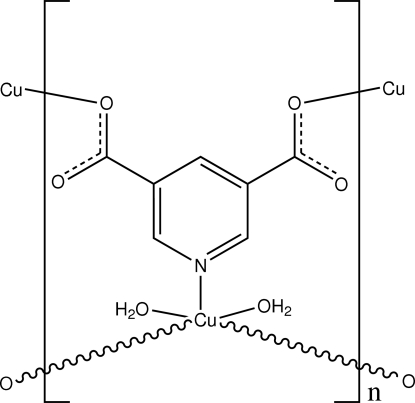


## Experimental

### 

#### Crystal data

[Cu(C_7_H_3_NO_4_)(H_2_O)_2_]
*M* *_r_* = 264.68
Monoclinic, 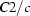

*a* = 10.1285 (16) Å
*b* = 12.0669 (19) Å
*c* = 7.2770 (11) Å
β = 101.584 (2)°
*V* = 871.3 (2) Å^3^
*Z* = 4
Mo *K*α radiation
μ = 2.52 mm^−1^
*T* = 298 K
0.23 × 0.18 × 0.07 mm

#### Data collection

Bruker APEXII 1000 CCD area-detector diffractometer
Absorption correction: multi-scan (*SADABS*; Sheldrick, 2004 ▶) *T*_min_ = 0.588, *T*_max_ = 0.840
2751 measured reflections
1003 independent reflections
892 reflections with *I* > 2σ(*I*)
*R*_int_ = 0.024

#### Refinement

*R*[*F* ^2^ > 2σ(*F* ^2^)] = 0.048
*wR*(*F* ^2^) = 0.148
*S* = 1.00
1003 reflections
70 parameters
H-atom parameters constrained
Δρ_max_ = 1.31 e Å^−3^
Δρ_min_ = −0.48 e Å^−3^

### 

Data collection: *APEX2* (Bruker, 2004 ▶); cell refinement: *SAINT* (Bruker, 2004 ▶); data reduction: *SAINT*; program(s) used to solve structure: *SHELXS97* (Sheldrick, 2008 ▶); program(s) used to refine structure: *SHELXL97* (Sheldrick, 2008 ▶); molecular graphics: *SHELXTL* (Sheldrick, 2008 ▶); software used to prepare material for publication: *SHELXTL*.

## Supplementary Material

Crystal structure: contains datablocks I, global. DOI: 10.1107/S1600536809013889/xu2500sup1.cif

Structure factors: contains datablocks I. DOI: 10.1107/S1600536809013889/xu2500Isup2.hkl

Additional supplementary materials:  crystallographic information; 3D view; checkCIF report

## Figures and Tables

**Table 1:** Selected geometric parameters (Å, °)

Cu1—O1*W*	1.964 (4)
Cu1—N1^i^	2.149 (4)
Cu1—O1	2.236 (3)

Symmetry codes: (i) 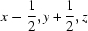; (ii) 
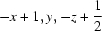
.

**Table 2:** Hydrogen-bond geometry (Å, °)

*D*—H⋯*A*	*D*—H	H⋯*A*	*D*⋯*A*	*D*—H⋯*A*
O1*W*—H1*WA*⋯O1^iii^	0.85	2.53	3.377 (5)	178
O1*W*—H1*WB*⋯O2^iv^	0.85	2.21	3.052 (5)	171

Symmetry codes: (iii) 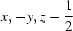; (iv) 
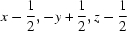
.

**Fig. 1.**
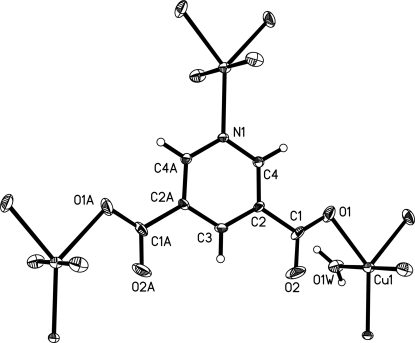
The molecular structure of the title complex with displacement ellipsoids drawn at the 30% probability level [symmetry code: (A) -x + 2, y, 1/2 - z].

**Fig. 2.**
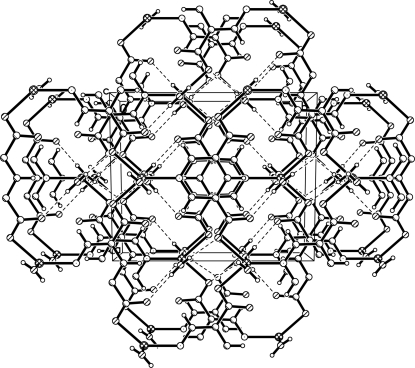
The crystal packing diagram of the title compound, viewed along the c axis.

## supplementary crystallographic information

### Comment

Over the past few years, much progress has been made toward the building of supramolecular structures with metal–organic compounds (Hou *et al.*, 2004). To get designed their intriguing frameworks and properties, an enormous amount of research is being focused in using versatile organic ligands and functional metal ions to construct the novel polymers (Chang *et al.*, 2005). The role of organic carboxylic acid ligand in synthesis such materials are of great interest. Here we report the hydrothermal synthesis and structure characterization of the title compound which is the isomorphism with Co^II^ complex reported in the previous literature (Whitfield *et al.*, 2001; Plater *et al.*, 1998).

The title compound crystallizes in space group *C*2/*c*. As illustrated in Fig. 1, in the asymmetric unit of it there is only one crystalographically distinct Cu^II^ ions which is coordinated by four O atoms and one N atom with the bond distance Cu—O 2.236 (3) and 2.236 (3) Å and Cu—N 2.149 (4) Å. The 3,5-PDA ligand acts as a tridentate ligand and bridges three equivalent Cu atoms with the Cu···Cu 7.877 Å. The O2 atom of each carboxylate group is terminal and oriented to the Cu1 atom with the Cu1···O2 distance 2.655 Å which are slightly larger than the Co^II^ isomorphism (Co···O_T_ 2.433 Å). A two-dimensional layer structure is thus constructed in the *ab* plane with openings along the c direction (Fig. 2). Hydrogen bonds are formed between coordinated water molecules and the carboxylate O atoms of adjacent layers (O1W···O1 3.377 (5) Å, O1W···O2 3.052 (5) Å) which furtherly connect the two-dimensional layers to a three-dimensional architecture. The shortes distance between Cu ions in the layers is 5.314 (2) Å.

### Experimental

The compound was synthesized by heating a mixture of Cu(CH_3_COO)_2_ (0.25 mmol, 0.05 g), 3,5-pyridinedicarboxylic acid (0.25 mmol, 0.0418 g), CH_3_OH (5 ml) and H_2_O (5 ml) in a Teflon-lined autoclave (25 ml) at 150 °C for 3 d. Green crystals of the title compound appeared after cooling to room temperature.

### Refinement

The water H atoms were placed in chemically sensible positions on the basis of hydrogen bonding, and were refined with distance restraint O—H = 0.85 Å. Other H atoms were placed in calculated positions and were refined in riding mode with C—H = 0.93 Å. *U*_iso_(H) = 1.2*U*_eq_(C,O).

### Crystal data

**Table:**

[Cu(C_7_H_3_NO_4_)(H_2_O)_2_]	*F*(000) = 532
*M**_r_* = 264.68	*D*_x_ = 2.018 Mg m^−^^3^
Monoclinic, *C*2/*c*	Mo *K*α radiation, λ = 0.71073 Å
Hall symbol: -C 2yc	Cell parameters from 2266 reflections
*a* = 10.1285 (16) Å	θ = 2.7–28.3°
*b* = 12.0669 (19) Å	µ = 2.52 mm^−^^1^
*c* = 7.2770 (11) Å	*T* = 298 K
β = 101.584 (2)°	Block, green
*V* = 871.3 (2) Å^3^	0.23 × 0.18 × 0.07 mm
*Z* = 4	

### Data collection

**Table:**

Bruker APEXII 1000 CCD area-detector diffractometer	1003 independent reflections
Radiation source: fine-focus sealed tube	892 reflections with *I* > 2σ(*I*)
graphite	*R*_int_ = 0.024
φ and ω scans	θ_max_ = 28.3°, θ_min_ = 2.7°
Absorption correction: multi-scan (*SADABS*; Sheldrick, 2004)	*h* = −13→12
*T*_min_ = 0.588, *T*_max_ = 0.840	*k* = −15→12
2751 measured reflections	*l* = −9→9

### Refinement

**Table:**

Refinement on *F*^2^	Primary atom site location: structure-invariant direct methods
Least-squares matrix: full	Secondary atom site location: difference Fourier map
*R*[*F*^2^ > 2σ(*F*^2^)] = 0.048	Hydrogen site location: inferred from neighbouring sites
*wR*(*F*^2^) = 0.148	H-atom parameters constrained
*S* = 1.00	*w* = 1/[σ^2^(*F*_o_^2^) + (0.108*P*)^2^ + 2.2514*P*] where *P* = (*F*_o_^2^ + 2*F*_c_^2^)/3
1003 reflections	(Δ/σ)_max_ < 0.001
70 parameters	Δρ_max_ = 1.31 e Å^−^^3^
0 restraints	Δρ_min_ = −0.48 e Å^−^^3^

### Special details

**Table:**

Geometry. All e.s.d.'s (except the e.s.d. in the dihedral angle between two l.s. planes) are estimated using the full covariance matrix. The cell e.s.d.'s are taken into account individually in the estimation of e.s.d.'s in distances, angles and torsion angles; correlations between e.s.d.'s in cell parameters are only used when they are defined by crystal symmetry. An approximate (isotropic) treatment of cell e.s.d.'s is used for estimating e.s.d.'s involving l.s. planes.
Refinement. Refinement of *F*^2^ against ALL reflections. The weighted *R*-factor *wR* and goodness of fit *S* are based on *F*^2^, conventional *R*-factors *R* are based on *F*, with *F* set to zero for negative *F*^2^. The threshold expression of *F*^2^ > σ(*F*^2^) is used only for calculating *R*-factors(gt) *etc*. and is not relevant to the choice of reflections for refinement. *R*-factors based on *F*^2^ are statistically about twice as large as those based on *F*, and *R*- factors based on ALL data will be even larger.

### Fractional atomic coordinates and isotropic or equivalent isotropic displacement parameters (Å^2^)

**Table:**

	*x*	*y*	*z*	*U*_iso_*/*U*_eq_	
Cu1	0.5000	0.16047 (5)	0.2500	0.0252 (3)	
O1W	0.4771 (4)	0.1611 (2)	−0.0245 (5)	0.0387 (8)	
H1WA	0.5216	0.1161	−0.0782	0.046*	
H1WB	0.4226	0.2065	−0.0896	0.046*	
O1	0.6503 (3)	0.0232 (3)	0.2667 (5)	0.0387 (8)	
O2	0.7647 (4)	0.1761 (3)	0.2777 (7)	0.0573 (12)	
C1	0.7562 (4)	0.0748 (4)	0.2691 (6)	0.0289 (9)	
C2	0.8837 (3)	0.0104 (3)	0.2610 (5)	0.0214 (7)	
C3	1.0000	0.0672 (4)	0.2500	0.0227 (10)	
H3A	1.0000	0.1443	0.2500	0.027*	
C4	0.8885 (3)	−0.1037 (3)	0.2620 (5)	0.0227 (8)	
H4A	0.8109	−0.1426	0.2713	0.027*	
N1	1.0000	−0.1614 (3)	0.2500	0.0215 (9)	

### Atomic displacement parameters (Å^2^)

**Table:**

	*U*^11^	*U*^22^	*U*^33^	*U*^12^	*U*^13^	*U*^23^
Cu1	0.0257 (4)	0.0176 (4)	0.0328 (4)	0.000	0.0073 (3)	0.000
O1W	0.049 (2)	0.0299 (18)	0.0370 (17)	0.0036 (12)	0.0074 (15)	−0.0021 (12)
O1	0.0211 (15)	0.0452 (19)	0.0507 (19)	0.0075 (12)	0.0097 (13)	−0.0097 (15)
O2	0.040 (2)	0.0264 (19)	0.101 (4)	0.0149 (14)	0.005 (2)	−0.0080 (18)
C1	0.0189 (19)	0.029 (2)	0.036 (2)	0.0121 (15)	−0.0006 (15)	−0.0074 (16)
C2	0.0162 (16)	0.0185 (17)	0.0293 (17)	0.0049 (12)	0.0043 (14)	−0.0009 (14)
C3	0.022 (3)	0.013 (2)	0.032 (3)	0.000	0.001 (2)	0.000
C4	0.0135 (16)	0.0187 (18)	0.0352 (19)	−0.0015 (12)	0.0033 (14)	−0.0009 (14)
N1	0.017 (2)	0.013 (2)	0.035 (2)	0.000	0.0062 (18)	0.000

### Geometric parameters (Å, °)

**Table:**

Cu1—O1W^i^	1.964 (4)	C1—C2	1.518 (5)
Cu1—O1W	1.964 (4)	C2—C4	1.378 (5)
Cu1—N1^ii^	2.149 (4)	C2—C3	1.379 (4)
Cu1—O1^i^	2.236 (3)	C3—C2^iii^	1.379 (4)
Cu1—O1	2.236 (3)	C3—H3A	0.9300
O1W—H1WA	0.8500	C4—N1	1.344 (4)
O1W—H1WB	0.8500	C4—H4A	0.9300
O1—C1	1.238 (5)	N1—C4^iii^	1.344 (4)
O2—C1	1.226 (5)	N1—Cu1^iv^	2.149 (4)
			
O1W^i^—Cu1—O1W	179.54 (17)	O2—C1—C2	117.5 (4)
O1W^i^—Cu1—N1^ii^	89.77 (8)	O1—C1—C2	118.9 (4)
O1W—Cu1—N1^ii^	89.77 (8)	C4—C2—C3	117.8 (3)
O1W^i^—Cu1—O1^i^	89.90 (13)	C4—C2—C1	122.8 (3)
O1W—Cu1—O1^i^	90.44 (13)	C3—C2—C1	119.4 (4)
N1^ii^—Cu1—O1^i^	137.80 (9)	C2—C3—C2^iii^	120.4 (5)
O1W^i^—Cu1—O1	90.44 (13)	C2—C3—H3A	119.8
O1W—Cu1—O1	89.90 (13)	C2^iii^—C3—H3A	119.8
N1^ii^—Cu1—O1	137.80 (9)	N1—C4—C2	123.2 (3)
O1^i^—Cu1—O1	84.40 (18)	N1—C4—H4A	118.4
Cu1—O1W—H1WA	120.0	C2—C4—H4A	118.4
Cu1—O1W—H1WB	120.0	C4^iii^—N1—C4	117.6 (4)
H1WA—O1W—H1WB	120.0	C4^iii^—N1—Cu1^iv^	121.2 (2)
C1—O1—Cu1	101.9 (3)	C4—N1—Cu1^iv^	121.2 (2)
O2—C1—O1	123.7 (4)		

Symmetry codes: (i) −*x*+1, *y*, −*z*+1/2; (ii) *x*−1/2, *y*+1/2, *z*; (iii) −*x*+2, *y*, −*z*+1/2; (iv) *x*+1/2, *y*−1/2, *z*.

### Hydrogen-bond geometry (Å, °)

**Table:**

*D*—H···*A*	*D*—H	H···*A*	*D*···*A*	*D*—H···*A*
O1W—H1WA···O1^v^	0.85	2.53	3.377 (5)	178
O1W—H1WB···O2^vi^	0.85	2.21	3.052 (5)	171

Symmetry codes: (v) *x*, −*y*, *z*−1/2; (vi) *x*−1/2, −*y*+1/2, *z*−1/2.
